# Lorcaserin Inhibit Glucose-Stimulated Insulin Secretion and Calcium Influx in Murine Pancreatic Islets

**DOI:** 10.3389/fphar.2021.761966

**Published:** 2021-11-05

**Authors:** Muhan Jing, Shanshan Wang, Ding Li, Zeyu Wang, Ziwen Li, Yichen Lu, Tong Sun, Chen Qiu, Fang Chen, Haijuan Yu, Wei Zhang

**Affiliations:** ^1^ School of Basic Medical Sciences, Nanjing Medical University, Nanjing, China; ^2^ Laboratory Animal Center, Department of Science and Technology, Nanjing University of Chinese Medicine, Nanjing, China; ^3^ Department of Forensic Medicine, School of Basic Medical Sciences, Nanjing Medical University, Nanjing, China; ^4^ Key Laboratory of Human Functional Genomics of Jiangsu Province, Nanjing Medical University, Nanjing, China; ^5^ Department of Biochemistry and Molecular Biology, School of Basic Medical Sciences, Nanjing Medical University, Nanjing, China; ^6^ Department of Obstetrics, Traditional Chinese Medicine Hospital of Jingning, Nanjing, China

**Keywords:** lorcaserin, glucose-stimulated insulin secretion, beta cell, 5-HT 2C R, obesity, type 2 diabetes mellitus, Ca^2+^

## Abstract

Lorcaserin is a serotonergic agonist specific to the 5-hydroxytryptamine 2c receptor (5-HT_2C_R) that is FDA approved for the long-term management of obesity with or without at least one weight-related comorbidity. Lorcaserin can restrain patients’ appetite and improve insulin sensitivity and hyperinsulinemia mainly through activating 5-HT_2C_R in the hypothalamus. It is known that the mCPP, a kind of 5-HT_2C_R agonist, decreases plasma insulin concentration in mice and previous research in our laboratory found that mCPP inhibited glucose-stimulated insulin secretion (GSIS) by activating 5-HT_2C_R on the β cells. However, the effect of lorcaserin on GSIS of pancreatic β cell has not been studied so far. The present study found that 5-HT_2C_R was expressed in both mouse pancreatic β cells and β-cell–derived MIN6 cells. Dose-dependent activation of 5-HT_2C_R by lorcaserin suppressed GSIS and SB242084 or knockdown of 5-HT_2C_R abolished lorcaserin’s effect *in vitro*. Additionally, lorcaserin also suppressed GSIS in high-fat diet (HFD)-fed mice in dose-dependent manner. Lorcaserin did not change insulin synthesis ATP content, but lorcaserin decrease cytosolic free calcium level [(Ca^2+^)i] in MIN6 cells stimulated with glucose and also inhibit insulin secretion and (Ca^2+^)i in MIN6 treated with potassium chloride. Furthermore, stimulation with the L-type channel agonist, Bay K8644 did not restore GSIS in MIN6 exposed to lorcaserin. Lorcaserin inhibits the cAMP generation of MIN6 cells and pretreatment with the Gα i/o inhibitor pertussis toxin (PTX), abolished lorcaserin-induced suppression of GSIS in β cells, while membrane-permeable cAMP analogue db-cAMP had same effect as PTX. These date indicated lorcaserin coupled to PTX-sensitive Gα i/o proteins in β cells reduced intracellular cAMP level and Ca^2+^ influx, thereby causing GSIS dysfunction of β cell. These results highlight a novel signaling mechanism of lorcaserin and provide valuable insights into the further investigation of 5-HT_2C_R functions in β-cell biology and it also provides guidance for the clinical application of lorcaserin.

## Introduction

The prevalence of obesity has become a major global health problem in both adults and adolescents (Eva et al., 2018). About 2 billion people are overweight in the worldwide and one third of them are obese (Lim et al., 2012). On the other hand, obesity, a major risk factor for type 2 diabetes mellitus (T2DM), could lead to the dysfunction of β cell in the pancreas, which is characterized by abnormalities in insulin synthesis and secretion (Ashcroft and Rorsman, 2012; Fu et al., 2013; Zhang et al., 2019; Lei et al., 2021). But the mechanism which leads to the onset of type 2 diabetes is still elusive.

5-hydroxytryptamine 2C receptor (5-HT_2C_R), a member of the super-family of G protein-coupled receptor (GPCR), belongs to the subfamily of serotonin receptors. 5-HT_2C_R has a widespread distribution in central nervous system (CNS) (Clemett et al., 2000). 5-HT_2C_R plays an important role in energy homeostasis and a wide variety of neuropsychiatric diseases, including eating disorders, drug addiction, schizophrenia, substance abuse, obsessive compulsive disorder, and epilepsy (Palacios et al., 2017; De Deurwaerdere et al., 2020; Yao et al., 2021). 5-HT_2C_R knockout mice in hypothalamus produces insulin resistance and type 2 diabetes, with hyperphagia, obesity, hyperinsulinemia, and impaired glucose tolerance (Zhou et al., 2007; Xu et al., 2008; Xu et al., 2010), where 5-HT_2C_R agonists are effective in improving glucose tolerance and hyperinsulinemia in murine models of obesity and insulin resistance (Zhou et al., 2007; Higgins et al., 2015; Burke et al., 2017), suggesting that 5-HT_2C_R is involved in improving insulin sensitivity and hyperinsulinemia. The expression of 5-HT_2C_R was also detected in the pancreas, isolated islets and pancreatic β-cell line (Bonhaus et al., 1995; Zhang et al., 2013; Nagata et al., 2019) and was increased in the islets of db/db mouse, an animal model for type 2 diabetes (Zhang et al., 2013). 5-HT is synthesized and co-secreted along with insulin in pancreatic β cells, potentially acting as a local autocrine/paracrine signal on insulin secretion (Ohta et al., 2011) and Interferes with the synthesis of 5-HT synthesis in beta cells impairs insulin secretion (Kim et al., 2015). All these suggested that the 5-HT_2C_R may play a direct role in regulating islet β cells function. Previous research in our laboratory found that meta-chlorophenylpiperazine (mCPP), a 5-HT_2C_R agonist, could inhibit glucose-stimulated insulin secretion (GSIS) in isolated mouse islets and MIN6 cells and that a 5-HT_2C_R antagonist SB242084 could reverse the inhibitory effects on the secretion of insulin produced by mCPP (Zhang et al., 2013). It could be concluded that activation of 5-HT_2C_R in β cell can cause dysfunction of insulin secretion.

Lorcaserin was approved by the Food and Drug Administration (FDA) in 2012 for chronic weight management (Colman et al., 2012). Lorcaserin reduces appetite, improves glucose tolerance and ameliorates hyperinsulinism among obese with or without T2DM patients through 5-HT_2C_R on POMC neurons of hypothalamus (Apovian et al., 2016; Pi-Sunyer et al., 2016). Lorcaserin has proven efficacy and safety in the treatment of obesity and its weight-related comorbidities including type 2 diabetes (Bohula et al., 2018a; Bohula et al., 2018b). However, in 2020, FDA sent a drug safety communication (DSC) reporting that more cancer cases were diagnosed after lorcaserin treatment (*n* = 462; 7.7%) compared to those in placebo group (*n* = 423; 7.1%) in a randomized, double-blind, placebo controlled clinical trial (FDA, 2020), which differs from the peer-reviewed publication claiming that cancer numbers is 215 (3.59%) and 210 (3.50%) in patients taking lorcaserin and in placebo, respectively (Bohula et al., 2018b). It indicated that more research about the safety of lorcaserin was still needed. However, whether lorcaserin have the insulinostatic effect on pancreatic β cells remains unclear at present. In this study, we investigated the effect of lorcaserin on GSIS of pancreatic β cells *in vitro* and *in vivo*, and explore the possible underlying molecular mechanism.

## Materials and Methods

### Animals, Islet Isolation and Cell Culture

All animal experiments were performed in accordance with the guidelines and rules formulated by the Animal Care Committee of Nanjing University of Chinese Medicine. Male C57BL/6J (6–8 weeks old) were obtained from Model Animal Research Center of Nanjing University (Nanjing, China) and housed under standard conditions with ad libitum access to chow and water, following a 12:12 h light and dark cycle at 25°C.

Isolation and culture of islets were performed as previously described (Han et al., 2001; Al-Amily et al., 2019). MIN6 cells, a mouse pancreatic β-cell line, were grown in DMEM medium containing 15% FBS (Life Technologies Co., Grand Island, NY), 10 mM HEPES, 50 mM β-mercaptoethanol, 100 U/ml penicillin and 100 mg/ml streptomycin. The cells were cultured at 37°C in a humidified atmosphere containing 95% air and 5% CO_2_. For compounds prepared in DMSO, the final concentration in the culture medium was kept at less than 0.1%.

### *In Vivo* Study

To induce obesity, male C57BL/6J mice were fed a high-fat diet (HFD, D12492, 60% energy from fat; Research Diets Inc., New Brunswick, NJ, United States) for 12 weeks and then were injected intraperitoneally with lorcaserin (616202-92-7, Aladin, Shanghai, China) at a concentration of 2.5, 4 or 10 mg/kg once a day for 2 weeks based on previous researches (Burke et al., 2017; Patel et al., 2020). Chronic injection of lorcaserin in diet-induced obesity (DIO) mice received intraperitoneal injections of lorcaserin at 2.5, 4 or 10 mg/kg as described above, whereas acute injection were only received a single intraperitoneal administration of lorcaserin. After fasting [16 h for intraperitoneal glucose tolerance test (IPGTT), 6 h for intraperitoneal insulin tolerance test (IPITT)], mice in acute and chronic treatment groups were all injected intraperitoneally with lorcaserin. 45 min later, 1.2 mg/kg glucose or 1 U/kg insulin were injected intraperitoneally into the mice, and blood was sampled from tail vein immediately prior to lorcaserin treatment, immediately prior to glucose or insulin injection, and 15, 30, 60, 90 and 120 min following glucose or insulin administration. Blood glucose was analyzed using an AlphaTRAK glucometer (Abbott Animal Health) and plasma insulin measurements with ELISA test kits (Ezassay Biotech Co., Ltd., Shenzhen City, China).

### Immunofluorescence

To confirm the 5-HT_2C_R protein expression in pancreatic samples and MIN6 cells, immunofluorescence was performed as described previously (Schultz et al., 2020). Mouse pancreases were fixed in 4% paraformaldehyde for 24 h at 4°C, embedded in paraffin, and sectioned. The slices were blocked with 5% goat serum plus in PBS for 1 h and incubated with a mouse anti-5-HT_2C_R monoclonal antibody (1:100, sc-17797, Santa Cruz) and rabbit anti-insulin antibody (1:250, ab181547 Abcam, Cambridge) or rabbit anti-glucagon (1:250, ab92517, Abcam) and then incubated with Alexa Fluor 488–conjugated donkey anti-mouse IgG (1:1000, A21202, Thermo Fisher) and Alexa Fluor 594–conjugated anti-rabbit IgG (1:1000, A21207, Thermo Fisher) for 1 h at RT. To stain nuclear DNA, the cells were treated with DAPI (ab104139, Abcam) for 5 min at RT. Negative control included the absence of primary antibodies. Images were taken on a Leica DM2500 microscope.

MIN6 cells were cultured on a glass slide and fixed with 4% paraformaldehyde for 15 min, followed by permeabilization with 0.1% Triton X-100 for 30 min, and blocked with 5% BSA for 30 min at room temperature, then cells were incubated with primary antibodies, the second antibodies and DAPI as above. Images were visualized by an Olympus FV1200 confocal laser scanning microscope system.

### Insulin Secretion

MIN6 cells (4*10^4^ cells per well) and isolated islets (10 islets per well) were cultured in 48-well plate and changed the culture medium every 24 h. Following preincubation for 1 h in glucose-free Krebs–Ringer bicarbonate HEPES buffer (KRBH, 119 mM NaCl, 4.74 mM KCl, 2.54 mM CaCl 2,1.19 mM MgCl 2,1.19 mM KH 2 PO 4, 25 mM NaHCO 3, 10 mM HEPES, pH 7.4), the MIN6 cells or islets were treated for 1 h in KRBH buffer with low glucose (2 mmol/L for MIN6 cells, 3.3 mmol/L for islets) or high glucose (20 mmol/L for MIN6 cells, 16.7 mmol/L for islets) or high KCL (50 mM) with or without lorcaserin, SB242084(2901, Tocris Bioscience), Bay K8644 (B112, Sigma-Aldrich) or db-cAMP (HY-b0764, MedChem Express LLC). For experiments involving pertussis toxin (PTX, P7208, Sigma-Aldrich) treatment, MIN6 cells were pretreated with PTX (150 ng/ml) overnight in the culture medium as described previously (Parandeh et al., 2020). The supernatants were then obtained for determination of insulin concentration. Intracellular insulin contents were extracted in acid–ethanol solution [74% (vol./vol.) ethanol, 1.4% (vol./vol.) HCl] overnight at 4°C. The insulin levels were measured by radioimmunoassay (RIA) kit (North Biological Technology Research Institute of Beijing) as described previously.

### Cell Viability Assay

Cell viability was determined using the Cell Counting Kit-8 (CCK8) assay (Dojindo Laboratories). Briefly, MIN6 (4 * 10^4^ cells per well) or mouse islets (10 islets/per well) were seeded in 96-well dishes, and treated with different concentrations of lorcaserin for 12 h. Then, each well was supplemented with 10 μL CCK8 and incubated for another 2 h at 37°C. The optical density (OD) value of each well was measured at the wavelength of 450 nm.

### 5-HT_2C_R Knockdown by Short Interfering RNA (siRNA) Transfection

The sequence for 5-HT_2C_R siRNAs (5’-CUA UCA ACA AUG AGA AGA A dTdT-3’) is selected based on previous research in our laboratory (Zhang et al., 2013) and the 5-HT_2C_R siRNA(si5-HT_2C_R) or scrambled control siRNA (siSCR) were purchased from RuiBo company (Guangzhou, China). Cells were transfected with siRNA, using Lipofectamine 2000 (Invitrogen). Medium was changed after 24 h and cells were assayed for knock down after 48 h, and then subjected to GSIS as described above.

### Real-Time PCR Assay

Total RNA was extracted from cultured cells using TRIzol reagent (Invitrogen) and quantified with Nanodrop 2000 (Thermo Fisher Scientific). Complementary DNA synthesis was performed using total RNA and first stand cDNA synthesis kit using random primers (Roche). Real-time PCR was performed using the SYBR Green PCR Master Mix (Vazyme Biotech Co., Ltd., China) and LightCycler480 II Sequence Detection System (Roche). Relative mRNA levels were calculated using the 2^–ΔΔCt^ method and normalized to the expression of β-actin. The primers sequences were shown as follows: 5-HT_2C_R froward (5′-GTT​CAA​TTC​GCG​GAC​TAA​GG-3′) and reverse (5′-TCA CGA ACA CTT TGCTTT CG-3′), Ins-1 forward (5′-CAC​TTC​CTA​CCC​CTG​CTG​G-3′) and reverse (5′-ACC​ACA​AAG​ATG​CTG​TTT​GAC​A-3′), Ins-2 forward (5′-GC TTCTTCTACAC ACCCATGTC-3′) and reverse (5′-AGC​ACT​GAT​CTA​CAA​TGC CAC-3′), β-actin forward (5′-AGG​CCA​ACC​GTG​AAA​AGA​TG-3′) and reverse (5′-AGAGCATAGCCC TCGTAGATGG-3′).

### Western Blot Analysis

MIN6 cells were lysed in an ice-cold radio immunoprecipitation assay (RIPA) lysis buffer (Millipore). After protein content determination, 30 μg total protein were loaded into 8% SDS-PAGE and transferred onto polyvinylidene difluoride (PVDF) membranes (Millipore). The membrane was incubated with primary antibodies against a 5-HT_2C_R (1:500, sc-17797, Santa Cruz) and β-Tubulin (1:1000, 2146, Cell Signaling) overnight at 4°C. After washed with tris-buffered saline with Tween 20 (TBST) for three times, the membranes were incubated in secondary antibody for 1 h at room temperature. The protein bands were visualized with enhanced chemiluminescence kit (ECL, Sigma-Aldrich).

### ATP Assays

Intracellular ATP content was measured using an enhanced ATP assay kit (S0027, Beyotime Biotechnology) according to the manufacturer’s instructions. In briefly, cells were lysed using ATP lysate buffer and then centrifuged at 12,000 g for 10 min at 4°C. The supernatant was removed and mixed with dilution buffer containing luciferase. The relative light units were measured by Gemini EM (Molecular Devices, Sunnyvale, CA). A fresh standard curve was prepared and ATP content was calculated using the curve and normalized to the protein content as determined by the BCA assay (T9300A, Takara).

### Measurement of Cytosolic Free Calcium Level [(Ca^2+^)i]

MIN6 cells cultured in 3.5 cm glass-bottomed dishes at 1*10^5^/ml and loaded with 5 μM Fluo-4 AM (F312, Dojindo Laboratories) for 40 min at room temperature in the dark. After washing, the cells were left for 30 min to allow de-esterification of the dye in the cytosol and then the glass-bottomed dishes were placed on the stage and recorded using an Olympus FV1200 confocal laser scanning microscope system (Olympus). The relative fluorescence signals were measured at excitation wavelength of 488 nm and emission wavelength of 516 nm. The fluorescence images were collected and analyzed using a FluoView software (Version 5.0, Olympus America Inc.). The results were plotted as the change in fluorescence intensities (F/F0), where F is the observed fluorescence density, and F0 is the average value of initial fluorescence intensity of 2.0 mM glucose (Liu et al., 2020). These data were analyzed and shown as the area under the original curve (AUC) as previously described.

### Measurement of Intracellular cAMP

MIN6 cells were incubated in KRBH supplemented with 2 or 20 mM glucose for 1 h in the absence or presence of 50 μM lorcaserin. After incubation, the cells were washed with KRBH and stored in RIPA buffer containing HCl (100 mM) and IBMX (100 mM, HY-12318, MedChem Express LLC). The supernatants were saved for cAMP measurement by enzyme-linked immunosorbent assay (ELISA) according to the manufacturer’s instructions (D770001-0096, Sangon Biotech). Final cAMP concentrations were normalized to total protein which was determined with the BCA Protein Assay Kit (T9300A, Takara).

### Statistical Analysis

Comparisons were performed using Student’s t test between two groups or ANOVA followed by Tukey–Kramer s multiple comparisons test in multiple groups. Results are presented as means ± SEM. A *P*-value < 0.05 was considered to be statistically significant.

## Results

### 5-Hydroxytryptamine 2c Teceptor is Expressed in Mouse Islet β Cells and MIN6 Cells

**FIGURE 1:**
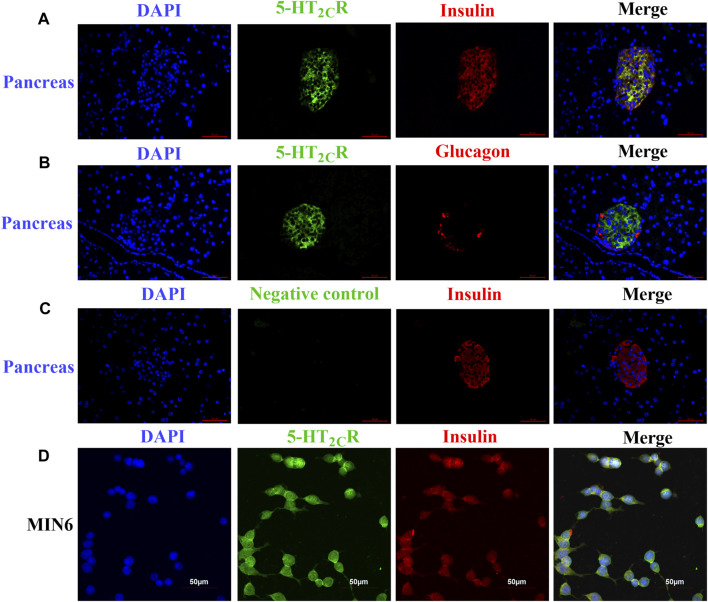
The expression of 5-HT_2C_R in pancreas of wild-type mice and MIN6 cells 5-HT_2C_R was colocalized with insulin in mouse pancreatic islets **(A)** and MIN6 cells **(D)**, whereas little to no 5-HT_2C_R was colocalized with glucagon **(B)**. The specificity of the anti-5-HT_2C_R antibody was validated by negative staining in pancreas of wild-type mice **(C)**. Scale bars: 50 μm.

Some research found 5-HT_2C_R is expressed in pancreas and islet of mouse and human (Bonhaus et al., 1995; Zhang et al., 2013; Nagata et al., 2019), although the precise expression is still controversial (Bennet et al., 2015; Bennet et al., 2016). Since previous results in our laboratory showed that 5-HT_2C_R was expressed in mouse islets and MIN6 by Western blot and qPCR (Zhang et al., 2013). Now the expression of 5-HT_2C_R were investigated by immunofluorescence assay. As shown in Figure 1, 5-HT_2C_R was detected in pancreatic islets of wild-type mice and colocalized with insulin (Figure 1A). In contrast, no 5-HT_2C_R immunofluorescence was colocalized with glucagon (Figure 1B). Expression of 5-HT_2C_R was also founded in mouse insulinoma MIN6 cells (Figure 1D).

### Lorcaserin Suppresses GSIS *in vitro*

**FIGURE 2:**
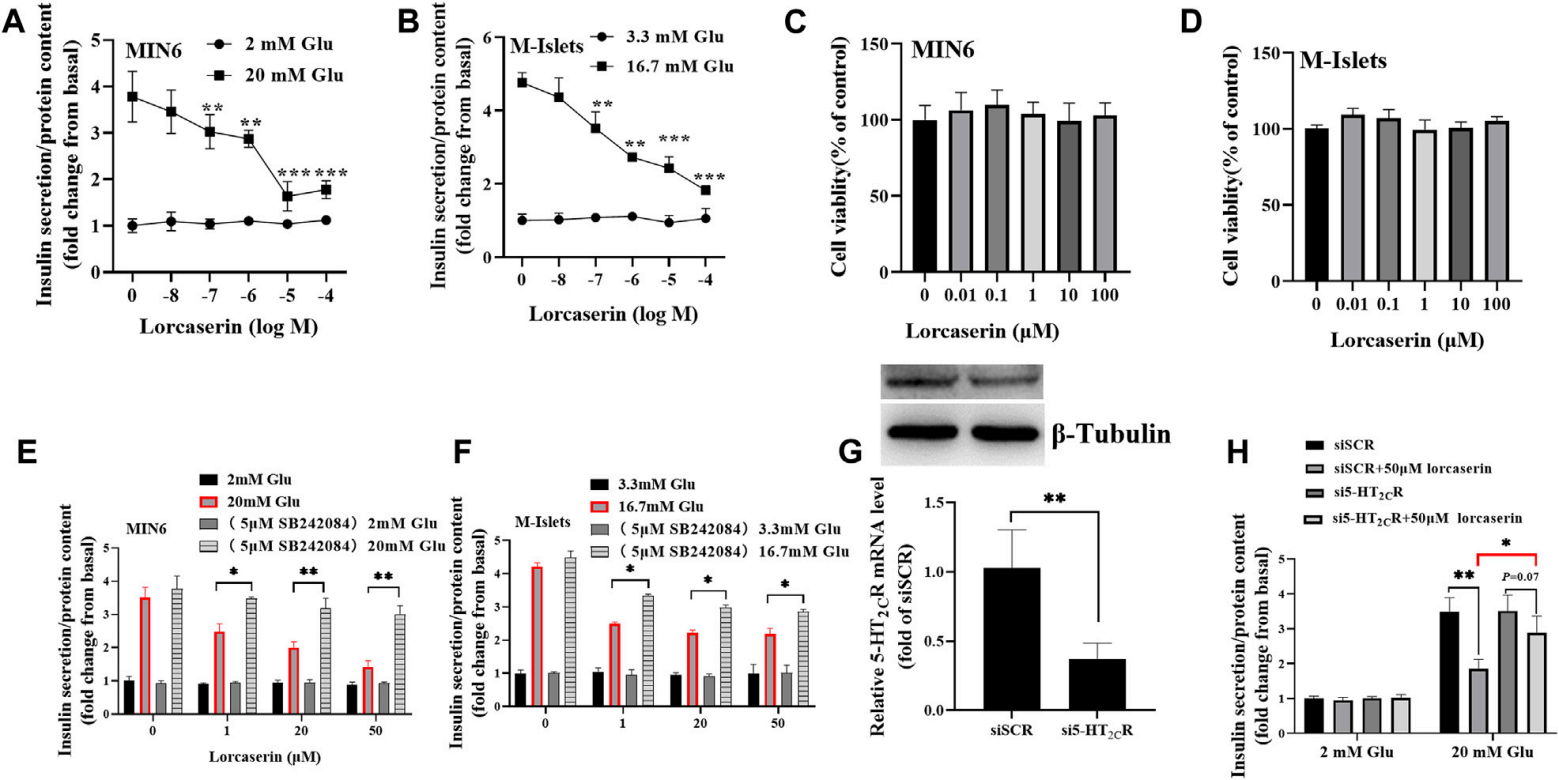
Lorcaserin inhibited glucose-stimulated insulin secretion of β cell. MIN6 cells **(A)** and mouse isolated islet **(B)** were pretreated with vehicle or 0.01–100 μM lorcaserin for 12 h and then then subjected to GSIS procedure. CCK8 assay were used to detect the cell viability of MIN6 **(C)** and mouse isolated islet **(D)**. MIN6 cells **(E)** and mouse isolated islet **(F)** were pretreated with vehicle, 1, 20 and 50 mM lorcaserin with or without 5-HT_2C_R antagonist SB242084 (5 μM) for 12 h and then subjected to GSIS procedure. **(G,H)** the effect of lorcaserin on GSIS in 5-HT_2C_R siRNA (si5-HT_2C_R) or scrambled control siRNA (siSCR) treated MIN6 cells. MIN6 cells (*n* = 3–6) and mouse isolated islet (*n* = 3–6), Glu, glucose; *p < 0.05, **p < 0.01.

As shown in Figure 2A, while the lorcasein, did not affect insulin release at 2 mM glucose, it dose-dependently inhibited GSIS (20 mM glucose) in MIN6 with a half-maximal inhibitory concentration (IC_50_) of 20.3 μM, which does not depend on its effect on cell viability (Figure 2C). A similar inhibitory action on GSIS and cell viability was observed in isolated mouse islets (Figures 2B,D). To further evaluate the role of 5-HT_2C_R on lorcaserin-dependent inhibition of GSIS, MIN6 cells were co-treated for 12 h with 1, 20 and 50 μM lorcaserin and 5-HT_2C_R specific antagonist SB242084 (5 μM). Preincubation with SBS22084 could significantly prevent the inhibitory effect of lorcaserin on insulin secretion in both MIN6 cells and mouse islet (Figures 2E,F). Furthermore, knockdown of 5-HT_2C_R by siRNA prevented the inhibitory effect of 50 μM lorcaserin on GSIS in MIN6 cells (Figures 2G,H). There were no differences in the total insulin content and the mRNA levels of Ins-1 and Ins-2 between lorcaserin-treated and control MIN6 cells (Supplementary Figures S1A,C) and isolated mouse islets (Supplementary Figures S1B,D), indicating that lorcaserin’s inhibitory effect on GSIS were not mediated by affecting insulin synthesis. All these data indicated that lorcaserin-mediated activation of 5-HT_2C_R is responsible for GSIS impairment in β cells.

### Lorcaserin Suppresses GSIS in Vivo

**FIGURE 3:**
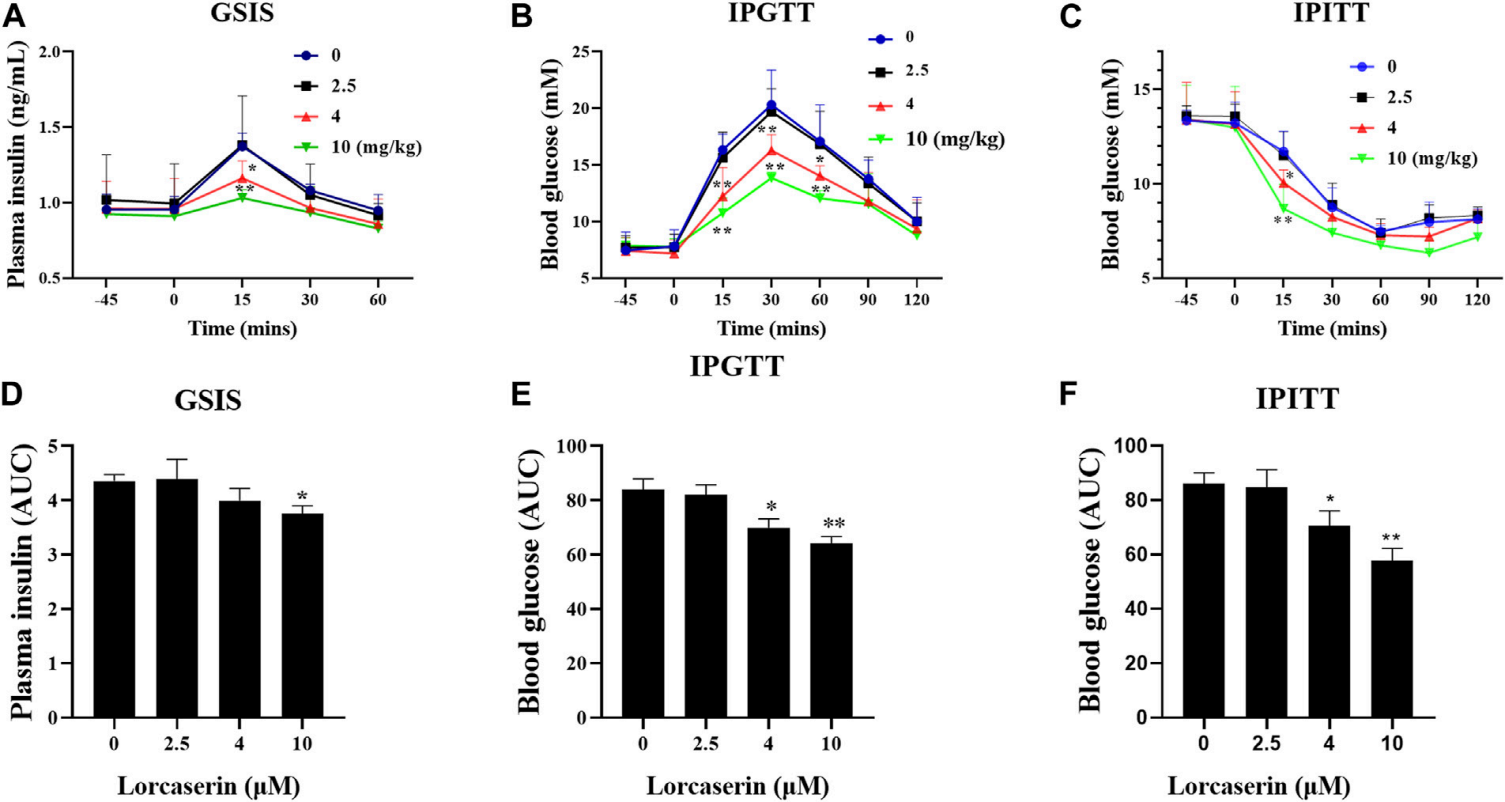
Acture injection of lorcaserin improved glucose tolerance and insulin sensitivity, but impaired GSIS. **(A,D)** Plasma insulin levels after an intraperitoneal injection of 1.2 mg/kg glucose **(A)** and the corresponding calculated AUC for insulin **(D)** in saline- or lorcasrein-treated mice (*n* = 5). **(B,E)** Blood glucose levels after an intraperitoneal injection of 1.2 mg/kg glucose **(B)** and the corresponding AUC for blood glucose **(E)** in saline- or lorcasrein-treated mice (*n* = 7–8). **(C,F)** Blood glucose levels after an intraperitoneal injection of 1 U/kg insulin **(C)** and the corresponding calculated AUC for blood glucose **(F)** in saline- or lorcasrein-treated mice (*n* = 5). Data are presented as mean ± SD. *p < 0.05, **p < 0.01 vs vehicle.

Lorcaserin is a weight-loss drug that can be used for management of obesity or obesity patients with type 2 diabetes (Bohula et al., 2018a; Bohula et al., 2018b). To investigate whether lorcaserin has effects on GSIS in vivo, male C57BL/6J mice were fed a high-fat diet for 12 weeks to induce obesity. As shown in Figure 3, there were no differences in plasma insulin concentration at between groups at baseline (*p* > 0.05). Glucose administration caused an increase in plasma insulin, which was inhibited by 4 and 10 mg/kg lorcaserin at 15 min after glucose administration (Figure 3A) and the average area under the curve (AUC) for plasma insulin in the acute injection of 10 mg/kg lorcaserin was significantly lower than that in the saline group (Figure 3D). But 2.5 mg/kg lorcaserin did not decrease plasma insulin and AUC values significantly (Figures 3A,D). Moreover similar results were obtained in DIO mice given chronic lorcaserin for 14 days (Supplementary Figures S2A,D). Moreover, acute and chronic treatment of lorcaserin improved glucose tolerance in a dose-dependent manner, and 4 and 10 mg/kg lorcaserin improved glucose tolerance significantly (Figures 3B,E and supplementary Figures S2B,E). Lorcaserin (4.0 and 10.0 mg/kg, IP) also significantly improved insulin sensitivity as measured with an insulin tolerance test (ITT) in DIO mice (Figures 3C,F and Supplementary Figures S2C,F).

### Lorcaserin Inhibits the (Ca^2+^)i Increase of MIN6 Cells

**FIGURE 4:**
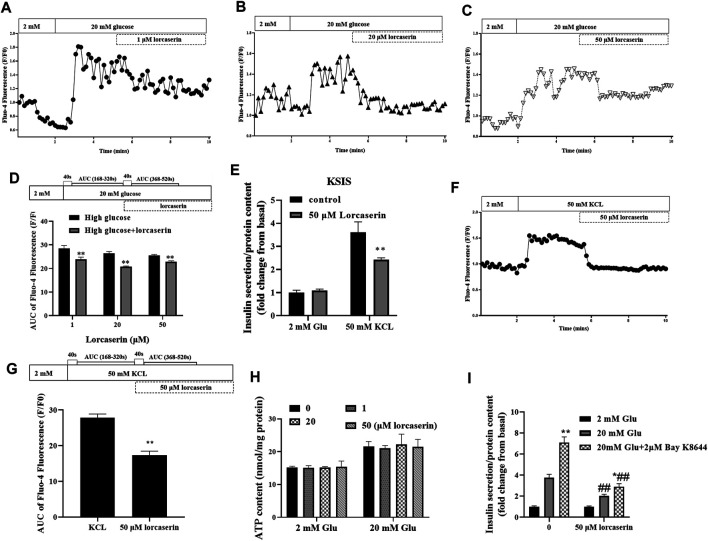
The inhibitory effect of lorcaserin on insulin secretion is mediated by selective decesae of (Ca^2+^)i in MIN6. **(A–D)** MIN6 cell were perfused with 2 or 20 mM glucose in the absence or presence of lorcaserin and the calcium influx were measured. **p < 0.01 vs high glucose. **(E–G)** The effect of 50 mM lorcaserin on insulin secretion and (Ca2^+^)i. under high KCL (50 mM) in MIN6 cells, **p < 0.01 vs high KCL. **(H)** MIN6 cells were pretreated with vehicle, 1, 20 and 50 mM lorcaserin under 2 or 20 mM glucose and then subjected to detecting the content of ATP in MIN6 cell. **(I)** MIN6 cells were exposed to 50 mM lorcaserin for 12 h and stimulated for 1 h with 2 mM glucose, 20 mM glucose, and 20 mM glucose with 2 μM Bay K8644, *p < 0.05 vs 20 mM glucose, **p < 0.01 vs 20 mM glucose, ##*p* < 0.01 lrocaserin+20 mM glucose vs 20 mM glucose and lrocaserin + Bay K8644 + 20 mM glucose vs 20 mM glucose Bay K8644 + 20 mM glucose. Data are presented as mean ± SD, *n* = 3–4.

Intracellular calcium influx is a key factor in the regulation of insulin release from pancreatic β cells (Seino, 2012). To evaluate whether lorcaserin suppresses GSIS *via* (Ca^2+^)i in β cells, we detected the (Ca^2+^)i in MIN6 cells using time-lapse laser scanning confocal microscopy. As shown in Figures 4A–C, (Ca^2+^)i was increased at 20 mmol/L glucose compared to 2 mmol/L glucose in MIN6 in all group. In contrast, (Ca^2+^)i was inhibited in MIN6 exposed to 1, 20 and 50 mM lorcaserin as indicated a smaller area under the curve between AUC _168–320s_ (41–200s after adding 20 mM gluocose)and AUC_368–520s_ (41–200s after adding lorcaserin) (Figure 4D).

According to the current consensus on the triggering pathway of GSIS, inhibition of plasma-K_ATP_ channels (ATP-sensitive K^+^ channels) by increased levels of ATP hyperpolarize the membrane of the β cell, stimulating the opening of voltage-gated L-type Ca^2+^ channels. Increased calcium entry through voltage-dependent L-type Ca^2+^ channels increases (Ca^2+^)i and trigger insulin release (Henquin, 2000; Seino, 2012).To further determine if the inhibition of (Ca^2+^)i by lorcaserin was due to inhibition of events upstream of membrane depolarization, we evaluated the (Ca^2+^)i and insulin secretion and after membrane depolarization induced by potassium chloride (KCL, 50 mM). Treatment with KCL stimulated both insulin secretion and (Ca^2+^)i in control group (Figures 4E–G). Lorcaserin treatment affect insulin secretion and (Ca^2+^)i that induced by KCL (Figures 4E–G); and our results showed that 1, 20 and 50 μM lorcaserin treatment did not change intracellular ATP content compared to that in the control group (Figure 4H). This suggests that lorcaserin may affect insulin secretion through the downstream of depolarization.

To test if lorcaserin inhibit insulin secretion by affecting [Ca^2+^]i through L-type Ca^2+^ channel, we measured GSIS in control and lorcasrin-exposed MIN6 treated with Bay K8644, a L-type Ca^2+^ channel agonist. Bay K8644 prolongs the time Bay K8644 potentiate insulin secretion by prolonging the opening time of L-type Ca^2+^ channels (Panten et al., 1985). MIN6 cells stimulated with Bay K8644 for an hour secreted more insulin at 20 mM glucose, but Bay K8644 did not reverse the inhibition of insulin secretion produced by lorcaserin, although lorcasrin and Bay K8644-exposed MIN6 secreted more insulin than lorcaserin-exposed MIN6 (Figure 4I). These data suggest that lorcaserin may affect L-type Ca^2+^ channel, thus disrupting the Ca^2+^-dependent assembly and/or exocytosis of insulin vesicles.

### Lorcaserin Inhibits the cAMP Generation of MIN6 Cells in a PTX-Sensitive Manner

**FIGURE 5:**
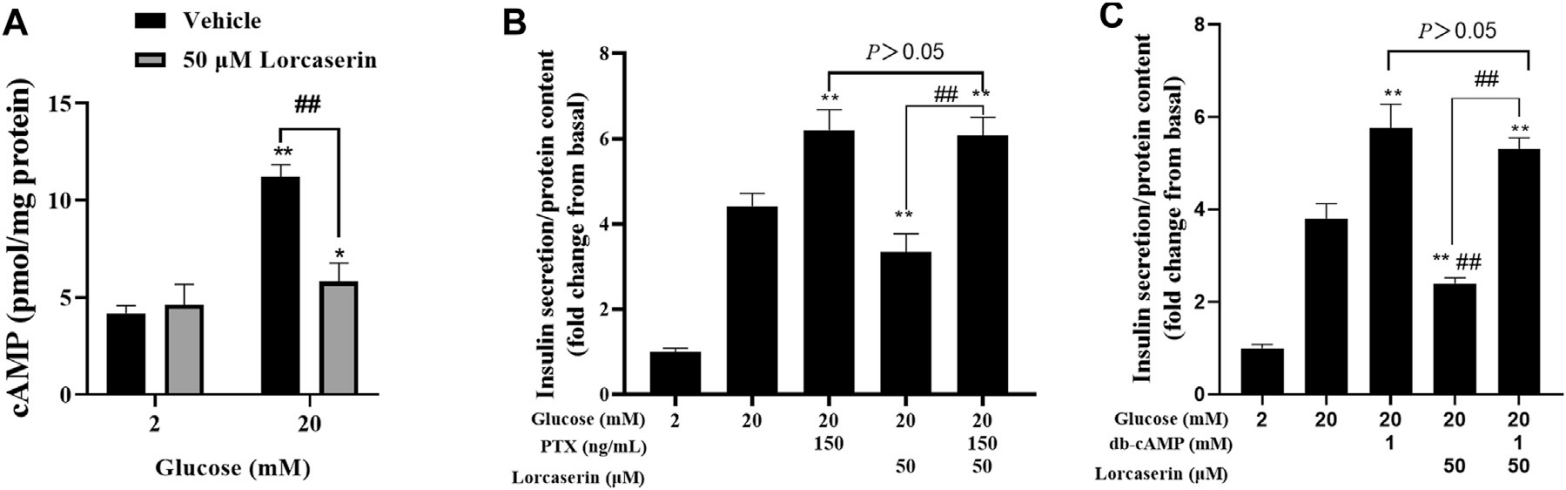
Lorcaserin inhibited insulin secretion by the cAMP-dependent manner. **(A)** MIN6 cells were pretreated with vehicle or 50 μM lorcaserin under 2 or 20 mM glucose for 12 h and then subjected to detecting the content of cellular cAMP. **(B, C)** Lorcaserin did not alter GSIS in the presence of Gαi/o inhibitor pertussis toxin (PTX, 150 ng/ml) in MIN6 cells in the presence of 20 mM glucose. **(C)** cAMP analogue db-cAMP averted the GSIS-diminishing effect of lorcaserin at 20 mM glucose. **p < 0.01 vs 20 mM glucos. Data are presented as mean ± SD, *n* = 3–6.

Activation of 5-HT_2C_R elevated the intracellular (Ca^2+^)i in SCN2.2 YC (a monoclonal rat SCN progenitor cell line) and CHO cells expressing 5-HT_2C_R primarily through Gα q to activate phospholipase C (PLC) pathway (Raymond et al., 2001; Cussac et al., 2008; Takeuchi et al., 2014), which is in contrast with the observed reduction of [Ca^2+^]i in β cells. But the 5-HT_2C_R also coupled to Gαi/o in *Xenopus* oocytes (Chen et al., 1994) and in HEK-293 cells (Alberts et al., 1999) and inhibited forskolin-stimulated cAMP production in AV12 cells (Lucaites et al., 1996). Many studies have confirmed that cAMP can affect insulin secretion by affecting calcium ion channels, calcium ion or vesicle transport (Kang et al., 2001; Seino, 2012). To evaluate whether lorcaserin suppresses GSIS *via* cAMP-dependent pathway in β cells, we detected the intracellular cAMP in the presence of lorcaserin in MIN6. The present study shows that 20 mM glucose increased the cAMP content and lorcaserin inhibits the cAMP generation of MIN6 cells under high glucose (Figure 5A) and preincubation of MIN6 with the Gαi/o inhibitor pertussis toxin (PTX, 150 ng/ml) completely reversed the inhibitory action of lorcaserin on GSIS (Figure 5B). To further evaluate cAMP-dependent pathways we performed insulin secretion in the presence of the membrane-permeable cAMP analogue db-cAMP in MIN6 cells. Lorcaserin no longer decreased GSIS (Figure 5C) when a cAMP reduction is counteracted by excess cAMP due to addition of db-cAMP (Figure 5C).

## Discussion

It has been shown that β-cell dysfunction is a leading pathophysiological sign of type 2 diabetes, which is characterized by abnormalities in synthesis and secretion of insulin ( Ashcroft and Rorsman, 2012; Fu et al., 2013; Zhang et al., 2019; Lei et al., 2021 ). Although these mechanisms for GSIS are well established, the underlying factors that mediate GSIS remain poorly understood.

GSIS is modulated by a number of factors, such as non-glucose nutrients (e.g., free fatty acids), hormones and neural inputs. Serotonin (5-HT) is a neurotransmitter, which can colocalize with insulin in secretory granules and cosecrete with insulin upon glucose-stimulation (Ohta et al., 2011; Kim et al., 2015). 5-HT produced contradictory results on the secretion of insulin; an inhibition (Lernmark, 1971; Zawalich et al., 2004) or a stimulation (Peschke et al., 1997) of GSIS has been observed in rodent islet. 5-HT receptors include seven distinct families (at least 14 different receptors) and virtually all 5-HT receptors are G protein-coupled with the exception of 5-HT3 receptors (Wyler et al., 2017). Almost all 5-HT receptors in human islets (Amisten et al., 2013; Bennet et al., 2015) and in rodent islets (Ohara-Imaizumi et al., 2013; Bennet et al., 2016; Nagata et al., 2019) were found and activating of different 5-HT receptors also has contradictory effect on GSIS of pancreatic β cells (Zhang et al., 2013; Bennet et al., 2015; Kim et al., 2015; Bennet et al., 2016). Previous research in our laboratory found that activating of 5-HT_2C_R on β cells by mCPP inhibited GSIS of mouse islets and MIN6 cells, which indicated that activation of 5-HT_2C_R leaded to dysfunction of insulin secretion (Zhang et al., 2013). In 2012, the FDA-approved lorcaserin as the first selective5-HT_2C_R agonist drug for the treatment of obesity. Approval was based on multiple phase III trials in obese population with or without type 2 diabetes (Smith et al., 2010; Fidler et al., 2011; Pedersen and Astrup, 2012), which was generally regarded as having a favorable tolerability and safety profile with a positive effect on obesity, insulin resistance and hyperinsulinemia of type 2 diabetes and the secondary outcome measures including blood pressure and total cholesterol, LDL-C, and triglycerides (Pedersen and Astrup, 2012; Aronne et al., 2014; Bohula et al., 2018b). However, none of all these studies focused on the effect of lorcaserin on GSIS of pancreatic β cells. Our functional data showed that lorcaserin could dose-dependently decrease GSIS in both MIN6 cells and mouse islets in high glucose conditions and this inhibitory effect is exerted *via* suppression of cellular cAMP generation by Gαi/o protein in PTX-sensitive manner, which was validated by PTX or db-cAMP treatment. Our results are thus in good agreement with the report showing that 5-HT_2C_R reduced cAMP generation by inhibiting adenylate cyclase activity through a Gαi/o-coupled protein (Chen et al., 1994; Lucaites et al., 1996; Alberts et al., 1999; Cussac et al., 2002), which is an important signaling molecule for GSIS (Seino, 2012; Amisten et al., 2013; Al-Amily et al., 2019; Parandeh et al., 2020). Our results also demonstrate lorcaserin did not changed the insulin content and the mRNA expression of ins1 and ins2 in MIN6 cells and mouse islets, which is consistent with previous studies on the effect of 5-HT on islets (Gagliard et al., 1974). In addition, lorcaserin did not affect cell viability of both MIN6 cells and mouse islets. All these results indicated lorcaserin’s inhibitory effect on GSIS is independent of its effect on insulin synthesis and cell viability.

Intracellular calcium influx is a key factor in the regulation of insulin release from pancreatic β cells (Seino, 2012). It has long been known that cAMP promotes the influx of calcium into β cells by voltage-dependent Ca^2+^ channel (Henquin and Nenquin, 2014). Activating of 5-HT_2A/2C_R by (2,5-dimethoxy-4-iodoamphetamine; DOI) inhibits Cav1.2 L-type Ca^2+^ currents in prefrontal pyramidal neruons (Day et al., 2002). Our results found that lorcaserin inhibited glucose-stimulated Ca^2+^ influx in MIN6 and stimulation with a L-type Ca^2+^ channel-specific agonist, Bay K8644, did not rescue the inhibition of insulin secretion in MIN6 exposed to lorcaserin and our results found that lorcaserin inhibited [Ca^2+^]i and the insulin secretion in MIN6 stimulated with a membrane depolarizing concentration of KCL. However, lorcaserin did not decrease the content of ATP under high glucose, suggesting that lorcaserin affect insulin-regulating mechanisms downstream from membrane depolarization, most likely affecting the voltage-gated calcium channels.

In vivo, we also detected the effect of lorcaserin on GSIS. Because previous research found that a single intraperitoneal injection of 2.5 mg/kg lorcaserin did not change GTT and ITT in DIO mice (Burke et al., 2017; Patel et al., 2020), but 4 mg/kg lorcaserin that was not sufficient to decrease food intake in DIO mice, affected both GTT and ITT through 5-HT_2C_R in POMC neruons of hypothalamus, and 10 mg/kg lorcaserin affected food intake, GTT and ITT, but 10 mg/kg lorcaserin did not changed the GSIS between 0 and 9 min (other time points were not detected) of DIO mice (Burke et al., 2017). In our research, we treated DIO mice with 2.5, 4 and 10 mg/kg lorcaserin and found that injection of lorcaserin (2.5, 4 and 10 mg/kg) produced a dose-dependent inhibition in GSIS, with 10 mg/kg significantly inhibition of GSIS at 15 min and AUC of insulin between 0 and 60 min in DIO mice compared to saline. We also found that 4 and 10 mg/kg lorcaserin significantly improved glucose tolerance and insulin sensitivity, which is consistent with the result of Burke, et al. (Burke et al., 2017). Moreover, These data indicate that high-dose lorcaserin inhibited GSIS, while low-dose did not, which may be related to activating 5-HT_2C_R mainly in hypothalamus by low-dose of lorcaserin and then improving insulin sensitivity, but having no obvious effect on 5-HT_2C_R on the islets. High-doses of lorcaserin could activate 5-HT_2C_R on islets of mice, leading to insulin secretion dysfunction, which also improving insulin sensitivity by activating the receptor on the hypothalamus. This can also explain the contradiction between the results of GSIS and GTT; and it may also be related to reducing hepatic glucose production and increasing glucose disposal through activating 5-HT_2C_R in the hypothalamus by lorcaserin (Thomsen et al., 2008; Xu et al., 2008; Higgins et al., 2015; Burke et al., 2017). It should be noted that the gene expression of 5-HT_2C_R decreased in the hypothalamus of DIO mice for 14 weeks compared with that of WT mice (Supplementary Figure S3), which was consistent with Schaffhauser et al. (2002), and lorcaserin treatment for 2 weeks reversed the expression of 5-HT_2C_R in DIO mice (Supplementary Figure S3). While the expression of 5-HT_2C_R were increased in the pancreas of DIO mice (which was consistent with our previous research detected in db/db mice (Zhang et al., 2013) and reversed by lorcaserin treatment (*p* = 0.08, Supplementary Figure S3).

In conclusion, our study revealed the detrimental effects of lorcaserin on the function of pancreatic β cell as well as the molecular mechanisms underneath, suggesting that there might be a higher risk to the function of β cells among obese patients who took lorcaserin as a weight-loss medicine. Our research also provides guidance for the clinical application of lorcaserin. Of course, our research also has certain limitations, there is a lack of human data on lorcaserin on GSIS, because lorcaserin has not yet been marketed in China. In addition to inhibiting adenylyl cyclases to inhibit insulin secretion by the PTX-dependent Gi/o pathway, PTX-dependent Gi/o also activates K^+^ channels to hyperpolarize the β cell and/or inhibit exocytosis to affect insulin release (Straub and Sharp, 2012). Further studies are needed to verify whether the inhibitory effect on insulin of lorcaserin is due to the hyperpolarization and/or exocytosis of β cells.

Further consideration, we have another idea about lorcaserin`s inhibitory effect on GSIS, which may have a protective effect on development of type 2 diabetes. In type 2 diabetes, because insulin sensitivity is reduced, the islet cells compensate by producing more insulin and long-term compensation is detrimental to the development of type 2 diabetes. If GSIS is inhibited in a high blood glucose state, it will lead to further compensation of islet cells and eventually accelerate the development of type 2 diabetes, such as glucocorticoids (Fichna and Fichna, 2017). Although lorcaserin inhibits GSIS by activating 5-HT_2C_R in the islets, lorcaserin improves glucose tolerance, ameliorates hyperinsulinism, reduces hepatic glucose production, increases glucose disposal and then improves high blood glucose through activating 5-HT_2C_R in the hypothalamus, which does not need islets to compensate to secrete more insulin to lowing blood glucose(Thomsen et al., 2008; Xu et al., 2008; Higgins et al., 2015; Apovian et al., 2016; Pi-Sunyer et al., 2016; Burke et al., 2017). The inhibition of GSIS by lorcaserin may be one of the reasons for its improvement of hyperinsulinemia in type 2 diabetes. In conclusion, the activation of 5-HT_2C_R receptors in the hypothalamus or in the islets by lorcaserin may be beneficial for type 2 diabetes.

## Data Availability

The original contributions presented in the study are included in the article/Supplementary Material further inquiries can be directed to the corresponding authors.
