# Supplementary material for: Lorcaserin Inhibit Glucose-Stimulated Insulin Secretion and Calcium Influx in Murine Pancreatic Islets
Source: Front Pharmacol. 2021 Nov 5;12:761966. doi: 10.3389/fphar.2021.761966 (PMC8602196; doi:10.3389/fphar.2021.761966)
Supplement: Supplementary file 1 [file DataSheet1.docx]

**Supplementary data**

**Supplementary Figures**

**Supplement Fig.1 Effects of lorcaserin on insulin synthesis of β cell.**

After 12h treatment under the indicated conditions, insulin content were detected in MIN6 cells (**A**) and mouse isolated islet (B). MIN6 cells (C) and mouse isolated islet (D) were treated under the indicated conditions for 12 h and the relative mRNA expression of Ins1 and Ins2 were determined. n = 3-6.

**Supplement Fig.2 Chronic injection of lorcaserin for two weeks improved glucose tolerance and insulin sensitivity, but impaired GSIS**

(A, D) Plasma insulin levels after an intraperitoneal injection of 1.2 mg/kg glucose (A) and the corresponding calculated AUC for insulin (D) in saline- or lorcasrein-treated mice (n=5). (B, E) Blood glucose levels after an intraperitoneal injection of 1.2 mg/kg glucose (B) and the corresponding AUC for blood glucose (E) in saline- or lorcasrein-treated mice (n=7-8). (C, F) Blood glucose levels after an intraperitoneal injection of 1U/kg insulin (C) and the corresponding calculated AUC for blood glucose (F) in saline- or lorcasrein-treated mice (n=5). Data are presented as mean ± SD. **P*< 0.05, ***P* < 0.01 vs vehicle

**Supplement Fig. 3 The expression of 5-HT2CR in hypothalamus and pancreas of mice**

Male C57BL/6J mice were fed a high-fat diet for 12 weeks and then were injected intraperitoneally by lorcaserin for two weeks. DIO, diet-induced obesity. Data are presented as mean ± SD. **P*< 0.05, ***P* < 0.01 vs vehicle, ## *P* < 0.01 vs DIO
